# ERTool: A Python Package for Efficient Implementation of the Evidential Reasoning Approach for Multi-Source Evidence Fusion

**DOI:** 10.34133/hds.0128

**Published:** 2024-08-05

**Authors:** Tongyue Shi, Liya Guo, Zeyuan Shen, Guilan Kong

**Affiliations:** ^1^ Institute of Medical Technology, Peking University Health Science Center, Beijing, China.; ^2^ National Institute of Health Data Science, Peking University, Beijing, China.; ^3^ Yau Mathematical Sciences Center, Tsinghua University, Beijing, China.; ^4^ Tandon School of Engineering, New York University, New York, NY, USA.; ^5^ Advanced Institute of Information Technology, Peking University, Hangzhou, China.

## Abstract

**Background:** Multi-source evidence fusion aims to process and combine evidence from different sources to support rational and reliable decision-making. The evidential reasoning (ER) approach is a helpful method to deal with information from multiple sources with uncertainty. It has been widely used in business analytics, healthcare management, and other fields for optimal decision-making. However, computerized implementation of the ER approach usually requires much expertise and effort. At present, some ER-based computerized tools, such as the intelligent decision system (IDS), have been developed by professionals to provide decision support. Nevertheless, IDS is not open source, and the user interfaces are a bit complicated for non-professional users. The lack of a free-to-access and easy-to-use computerized tool limits the application of ER. **Methods:** We designed and developed a Python package that could efficiently implement the ER approach for multi-source evidence fusion. Further, based on it, we built an online web-based system, providing not only real-time evidence fusion but also visualized illustrations of combined results. Finally, a comparison study between the Python package and IDS was conducted. **Results:** A Python package, ERTool, was developed to implement the ER approach automatically and efficiently. The online version of the ERTool provides a more convenient way to handle evidence fusion tasks. **Conclusions:** ERTool, compatible with Python 3 and can be installed through the Python Package Index at https://pypi.org/project/ERTool/, was developed to implement the ER approach. The ERTool has advantages in easy accessibility, clean interfaces, and high computing efficiency, making it a key tool for researchers and practitioners in multiple evidence-based decision-making. It helps bridge the gap between the algorithmic ER and its practical application and facilitates its widespread adoption in general decision-making contexts.

## Introduction

Decision-making refers to the process and logic through which we can choose between 2 or more alternatives. Due to the complexity of decision-making in different areas, a decision-maker would turn to all possible sources of information to make an informed decision based on facts rather than intuition. However, data collected from a single source often fail to achieve informative and reliable choices. Under this circumstance, multi-source evidence fusion emerges to comprehensively process and combine evidence from different sources to support reliable and accurate decision-making. In practical applications, multi-source evidence fusion technology plays a crucial role in business analytics, healthcare management, environmental risk assessment, equipment fault diagnosis, image processing, and other fields [1–6]. It leverages the independence and uniqueness of the evidence to enhance decision reliability and rationality. However, uncertainty is a big challenge in real-world applications. The evidential reasoning (ER) approach [7] is an important method to address uncertain decision-making problems by multi-source evidence fusion. It has widespread applications in various fields, such as medicine and engineering [2,8,9]. Developed from Dempster’s rule for combining 2 or more belief functions based on evidence from different sources, the ER approach can merge evidence and provide combined results for decision support [10–12]. This approach allows decision-makers to use a distributed format of belief degrees or probabilities to represent uncertainty in evidence. Notably, in medicine and healthcare, which have inherent uncertainty, the ER approach can play an important role in integrating multi-source clinical data and thus facilitate complex decision-making [13,14].

During the past 2 decades, the ER approach has been gradually developed and attracted attention from researchers in different decision-making fields under uncertainty. However, the computerized implementation of the approach is a bit complicated for non-professionals. In the literature, we can see that an intelligent decision system (IDS) [15] based on the ER approach has been developed, and it has gained wide applications. To use the ER-based IDS, users need to download and install the system locally, model decision problems visually, and manually input the evidence data related to each problem. There is a lack of open-source and easy-to-use tools to deal with multi-source evidence fusion problems in an intelligent and batch-processing mode. Notably, we have not yet seen such a free-to-access tool implemented using the ER approach in mainstream programming environments like Python. The lack of a publicly available and online ER-based tool limits the broader application of the approach, especially in areas requiring timely data analytics and rapid prototyping. Therefore, an open-source and free-to-access ER tool is strongly demanded to facilitate the utilization and application of the ER approach by researchers and practitioners in different areas. To fill the gap, we designed and developed an open-source and publicly available Python package to implement the ER approach.

The ERTool, a Python package designed to efficiently implement the ER approach, and its online version called ERTool Online are introduced in this paper. The main advantages of the ERTool lie in its free accessibility, clean interfaces, and high computing efficiency, making it an easy-to-use and efficient tool especially suited for dealing with decision-making problems with multi-source evidence or data. This toolkit makes a complex and multiple evidence-based decision-making process efficient, reliable, and visualized. The ERTool addresses some limitations of the existing ER-based IDS, lowers the barrier to using the ER approach, and makes it easier to be applied in a broader range of research and practice areas. We recognize the dynamic nature of technological advancements in ER and commit to continuous improvement of ERTool to ensure its alignment with the latest developments in the field. The remainder of this paper is structured as follows: design and development of the ERTool package are introduced in the “Design and Development” section; the “Use Instruction and Examples” section provides use instruction and examples; validation and comparison of the tool with IDS are described in the “Validation and Comparison” section; and the “Conclusion and Future Work” section summarizes with discussion and conclusions of this tool.

## Design and Development

### The ER approach

The ER approach was proposed by Yang and Singh [7] for handling multi-criteria or multi-attribute decision-making problems under uncertainty. In the context of multi-criteria decision-making, assessment or evaluation of a criterion is considered as one piece of evidence supporting an object, denoted using *y*.

Suppose there are *L* independent pieces of evidence *e_i_*(*i* = 1, …, *L*). If a common set of *N* mutually exclusive evaluation grades or propositions *θ_n_*(*n* = 1, …, *N*)(*θ_i_* ∩ *θ_j_* = ∅, *i* ≠ *j*) are used to evaluate each criterion of object *y*, then a frame of discernment, denoted by *Θ* = {*θ_n_*, *n* = 1, …, *N*}, is formed. If we use *P*(*Θ*) to denote the power set of *Θ*, which consists of 2*^N^* subsets of *Θ*, we get *P*(*Θ*) = {∅, *θ*_1_, ⋯, *θ_N_*, {*θ*_1_, *θ*_2_}, ⋯, {*θ*_1_, ⋯, *θ*_*N* − 1_}, *Θ*}.

In the ER approach, each piece of evidence or assessment of each criterion is represented by a set of belief degrees distributed on different propositions. Suppose an object *y* is assessed to a proposition *θ_n_* in the *i*th evidence *e_i_* with a belief degree *p*_*n*,*i*_. Evidence *e_i_* in the distributed format is represented as

$$e_{i}=\left\{\left(\theta_{n},p_{n,i}\right),n=1,\ldots ,N;\left(\Theta ,p_{\Theta ,i}\right)\right\}$$ (1)

where 0 ≤ *p*_*n*,*i*_ ≤ 1(*n* = 1, …, *N*) with $\sum_{n=1}^{N}p_{n,i}\leq 1$, $p_{\Theta ,i}=1-$$\sum_{n=1}^{N}p_{n,i}$ is used to denote the degree of ignorance, representing the belief degree that has not been assigned to any proposition in *Θ*.

Each piece of evidence can be assigned a weight *w_i_*(*i* = 1, …, *L*) according to the importance of corresponding criterion, and all weights are normalized to satisfy that $0\leq w_{i}\leq 1$ and $\sum_{i=1}^{L}w_{i}=1$. Let *m*_*n*, *i*_(*n* = 1, …, *N*; *i* = 1, …, *L*) be the basic probability mass that the object *y* being assessed to proposition *θ_n_* with the support of evidence *e_i_*, and *m*_*Θ*, *i*_ be the probability mass unassigned to any proposition after all the propositions have been considered for evidence *e_i_*. They are calculated as the following:

$$m_{n,i}=w_{i}p_{n,i}$$ (2)

$$m_{\Theta ,i}=1-\sum_{n=1}^{N}m_{n,i}=1-\sum_{n=1}^{N}w_{i}p_{n,i}$$ (3)

Here, *m*_*Θ*,*i*_ represents the uncertainty in the assessment of object *y* caused by evidence *e_i_*, and it is composed of 2 parts: ${\tilde{m}}_{\Theta ,i}$, a remaining probability mass unassigned to any proposition due to the incompleteness of evidence *e_i_*, and ${\bar{m}}_{\Theta ,i}$, a remaining probability mass unassigned to any proposition caused by the weight unassigned to evidence *e_i_*. Their calculations are as follows:

$$m_{\Theta ,i}={\tilde{m}}_{\Theta ,i}+\;{\bar{m}}_{\Theta ,i}$$ (4)

$${\tilde{m}}_{\Theta ,i}=w_{i}p_{\Theta ,i}=w_{i}\left(1-\sum_{n=1}^{N}p_{n,i}\right)$$ (5)

$${\bar{m}}_{\Theta ,i}=1-w_{i}$$ (6)

where ${\tilde{m}}_{\theta ,i}$ will be zero if evidence *e_i_* is complete and there is no ignorance in it.

Let *e*_(*i*)_ be the subset of the first *i* pieces of evidence with *e*_(*i*)_ = {*e*_1_, *e*_2_, …, *e_i_*}, *m*_*n*,*e*(*i*)_ be the probability mass defined as the degree to which all the *i* pieces of evidence support the hypothesis that the object *y* be assessed to proposition *θ_n_*, and *m*_*Θ*,*e*_(*i*)__ be the probability mass unassigned to any proposition after combining *e*_(*i*)_ due to uncertainty. Similarly, *m*_*Θ*,*e*_(*i*)__ is composed of 2 parts ${\tilde{m}}_{\Theta ,e_{\left(i\right)}}$ and ${\bar{m}}_{\Theta ,e_{\left(i\right)}}$ with

$$m_{\Theta ,e_{\left(i\right)}}={\tilde{m}}_{\Theta ,e_{\left(i\right)}}+{\bar{m}}_{\Theta ,e_{\left(i\right)}}$$ (7)

Given the above definitions, the recursive ER algorithm is described as follows:

$$\begin{array}{r} m_{n,e_{\left(i+1\right)}}=K_{e_{\left(i+1\right)}}\left[m_{n,e_{\left(i\right)}},m_{n,i+1},+,m_{n,e_{\left(i\right)}},\left({\tilde{m}}_{\Theta ,i+1}+{\bar{m}}_{\Theta ,i+1}\right)\right.\\ \;\left.+m_{n,i+1}\left({\tilde{m}}_{\Theta ,e_{\left(i\right)}}+{\bar{m}}_{\Theta ,e_{\left(i\right)}}\right)\right] \end{array}$$ (8)

$${\tilde{m}}_{\Theta ,e_{\left(i+1\right)}}=K_{e_{\left(i+1\right)}}\left({\tilde{m}}_{\Theta ,e_{\left(i\right)}}{\tilde{m}}_{\Theta ,i+1}+{\bar{m}}_{\Theta ,e_{\left(i\right)}}{\tilde{m}}_{\Theta ,i+1}+\right.\left.{\tilde{m}}_{\Theta ,e_{\left(i\right)}}{\bar{m}}_{\Theta ,i+1}\right)$$ (9)

$${\bar{m}}_{\Theta ,e_{\left(i\right)}}=K_{e_{\left(i+1\right)}}\left({\bar{m}}_{\Theta ,e_{\left(i\right)}}{\bar{m}}_{\Theta ,i+1}\right)$$ (10)

Kei+1=1−∑n=1N∑k=1k≠nNmn,eimk,i+1−1i=1,2,…,L−1 (11)

where *K*_*e*_(*i* + 1)__ is used as a normalizing factor to make $\sum_{n=1}^{N}$$m_{n,e_{\left(i+1\right)}}+m_{\Theta ,e_{\left(i+1\right)}}=1$. It is worth noting that *m*_*n*,*e*(1)_ = *m*_*n*,1_, ${\bar{m}}_{\Theta ,e\left(1\right)}={\bar{m}}_{\Theta ,1}=1-w_{1}$, and ${\tilde{m}}_{\Theta ,e_{\left(1\right)}}={\tilde{m}}_{\Theta ,1}=w_{1}\left(1\;-\;\sum_{n=1}^{N}p_{n,1}\right)$ .

Let *p_n_* be the final belief degree supporting the hypothesis that the object *y* is assessed to the *n*th proposition *θ_n_* after combining all the *L* pieces of evidence. The combined belief degree *p_n_* distributed on proposition *θ_n_* is given by

$$p_{n}=\frac{m_{n,e_{\left(L\right)}}}{1-{\bar{m}}_{\Theta ,e_{\left(L\right)}}},n=1,2\ldots ,N$$ (12)

$$p_{\Theta }=\frac{{\tilde{m}}_{\Theta ,e_{\left(L\right)}}}{1-{\bar{m}}_{\Theta ,e_{\left(L\right)}}}$$ (13)

where *p_Θ_* denotes the belief degree unassigned to any proposition after combining all the *L* pieces of evidence due to the uncertainty in all evidence.

Take the first 2 pieces of evidence *e*_1_ and *e*_2_ as examples. Use *m*_*n*,*e*_(2)__ to denote the probability mass that supports the proposition *θ_n_* after fusing the first 2 pieces of evidence *e*_1_ and *e*_2_. According to the above-described ER algorithm, the mathematical formulas for the combination of evidence *e*_1_ and *e*_2_ are as follows:

Ke2=1−∑n=1N∑k=1k≠nNmn,e1mk,2−1 (14)

$$\begin{array}{c} m_{n,e_{\left(2\right)}}=K_{e_{\left(2\right)}}\left[m_{n,e_{\left(1\right)}},m_{n,2},+,m_{n,e_{\left(1\right)}},\left({\tilde{m}}_{\Theta ,2}+{\bar{m}}_{\Theta ,2}\right)\right.\\ \;\left.+,m_{n,2},\left({\tilde{m}}_{\Theta ,e_{\left(1\right)}}+{\bar{m}}_{\Theta ,e_{\left(1\right)}}\right)\right] \end{array}$$ (15)

$${\tilde{m}}_{\Theta ,e_{\left(2\right)}}=K_{e_{\left(2\right)}}\left({\tilde{m}}_{\Theta ,e_{\left(1\right)}}{\tilde{m}}_{\Theta ,2}+\right.\left.{\tilde{m}}_{\Theta ,e_{\left(1\right)}}{\bar{m}}_{\Theta ,2}+{\bar{m}}_{\Theta ,e_{\left(1\right)}}{\tilde{m}}_{\Theta ,2}\right)$$ (16)

$${\bar{m}}_{\Theta ,e_{\left(2\right)}}=K_{e_{\left(2\right)}}\left({\bar{m}}_{\Theta ,e_{\left(1\right)}}{\bar{m}}_{\Theta ,2}\right)$$ (17)

$$p_{n,e_{\left(2\right)}}=\frac{m_{n,e_{\left(2\right)}}}{1-{\bar{m}}_{n,e_{\left(2\right)}}}$$ (18)

$$p_{\Theta ,e_{\left(2\right)}}=\frac{{\tilde{m}}_{\Theta ,e_{\left(2\right)}}}{1-{\bar{m}}_{n,e_{\left(2\right)}}}$$ (19)

The combined evidence after fusing the 2 pieces of evidence can be expressed as the following belief distribution:

$$e_{\left(2\right)}=\left\{\left(\theta_{n},p_{n,e_{\left(2\right)}}\right)n=1\ldots N\left(\Theta ,p_{\Theta ,e_{\left(2\right)}}\right)\right\}.$$ (20)

If pieces of evidence are equally important and the evidence weight is not considered in the combination process, the ER-based combination algorithm for any 2 pieces of evidence turns into the Dempster’s combination rule [10,16].

$$m_{n,e_{\left(2\right)}}=\frac{\sum_{B\cap C=\theta_{n}}m_{B,1}m_{C,2}}{1-\sum_{B\cap C=\emptyset \emptyset }m_{B,1}m_{C,2}},\;\theta_{n}\neq \emptyset \emptyset ,n=1,\cdots ,N.$$ (21)

Here, B and C are subsets of propositions in the discernment frame *Θ*, and *m*_*B*,1_ and *m*_*C*,2_ are probability mass assigned to B in the first evidence and C in the second evidence, respectively. Specifically, *m*_*B*,1_ = *p*_*B*,1_ and *m*_*C*,2_ = *p*_*C*,2_, where *p*_*B*,1_ and *p*_*C*,2_ are the degrees of belief in B supported by the first evidence and in C supported by the second evidence, respectively.

Compared to the ER approach, the evidence weight is not considered in the Dempster’s combination rule, and it assumes that all evidence holds equal importance. This assumption lacks universality. Besides, Dempster’s combination rule allocates all residual probability or belief degrees to the discernment frame, treating it as ignorance without distinguishing it between incompleteness and partial importance. In other words, Dempster’s combination rule considers uncertainty as a whole, while the ER approach divides uncertainty into one part caused by relative importance and the other part caused by incompleteness of the evidence [17]. Another limitation of Dempster’s combination rule is its failure to define the scenario where 2 pieces of evidence are in complete conflict, where each piece of evidence supports different propositions. When Dempster’s combination rule is applied to fuse evidence of high (or near complete) conflict, this lack of definition leads to a counterintuitive combined result. On the other hand, the Dempster’s combination rule can actually be used as a tool to validate the rationality and correctness of the ER approach.

### Programming implementation

The core purpose of ERTool is to implement the ER approach in Python. Our implementation follows a structured approach closely aligned with the theoretical underpinnings of the ER algorithm and optimized for clarity and computational efficiency. We implement the recursive ER algorithm by iterating through matrix calculation. The specific calculation procedures of the ER algorithm are shown in Algorithm 1, and its programming implementation is the er_algorithm() function in ERTool.

Input: (1) *W* is a one-dimensional array of floats. It represents the weights of all evidence. These weights are used in the algorithm to adjust the influence of each evidence. (2) *DBF* represents a 2-dimensional array of floats. It stands for Degrees of Belief and is one of the main inputs to the algorithm, representing the initial degree of belief in each proposition supported by each evidence. (3) *numOfEvidence*(*nE*) is an integer. It indicates the number of evidence to be combined. In the *DBF* array, it corresponds to the number of rows. (4) *numOfPropositions*(*nP*) is an integer, too. It indicates the number of propositions or evaluation grades. In the *DBF* array, it corresponds to the number of columns.

Output: (1) *B* (Array): Upon completion of the algorithm, the *B* array is updated with the final calculation results. It reflects the degree of belief in each proposition or evaluation grade for the object being assessed after combining all available evidence. The first *nP* members in the *B* array represent the degree of belief in each proposition after evidence fusion. The last member of the *B* array represents the belief degree unassigned to any proposition after evidence combination, and it denotes the overall uncertainty. (2) *False* (Boolean): It returns True if the algorithm successfully executes and completes all computations. If any error is encountered during execution (e.g., division by zero), it returns False.


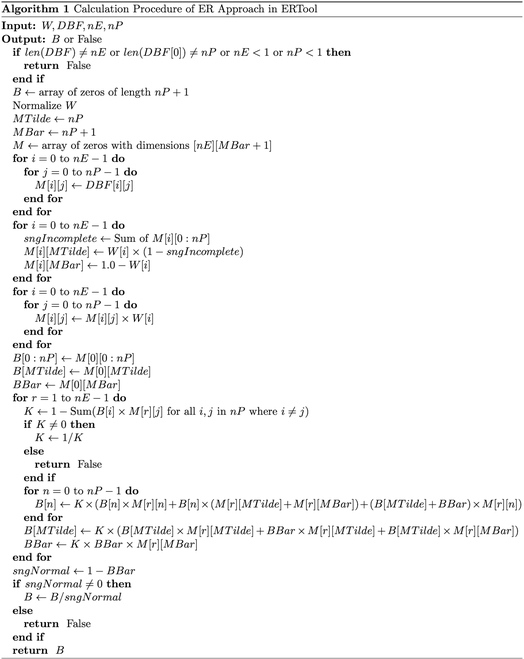


Initial setup and check: The algorithm begins with an essential check step, ensuring the integrity of input data. It checks the matching between input dimensions for the degree of belief (*DBF*) array and weight vector (*W*), returning an error if the inputs are not correctly aligned. Data structures and normalization: A crucial part of the algorithm is the initialization of output variables and normalization of input or intermediate variables. The array *B* designed to hold the final combined belief degrees is initialized by zero, and the weight vector *W* is normalized with a sum to be one, ensuring the proper scaling of influence from different sources. Matrix transformation and weight calculation: The core transformation involves converting the *DBF* array into a matrix *M*, which is then used for the calculation of combined belief degrees. Each element of *M* is adjusted based on the normalized weights, reflecting the contribution of each evidence source. Recursive combination: The strength of the ER algorithm lies in its recursive combination of evidence, a process implemented through a series of nested loops. This recursion is meticulously designed to ensure that all parameters related to evidence are combined, including their respective weights and incompleteness. After the recursive combination, the algorithm normalizes the combined belief degrees using an integrity factor. This step is crucial in ensuring the validity of the final belief degrees. Error handling: Throughout the algorithm, careful attention is given to error handling, especially for potential division by zero scenarios, ensuring robustness in all computational situations.

We paid attention to the efficiency and readability of coding in implementing ERTool. Python’s inherent readability and our structured coding approach make the ERTool a powerful and publicly accessible computational tool for researchers and practitioners in various fields.

### Functions in ERTool

We implemented the ER algorithm in Python as ERTool, and its current version has some functions. They are er_algorithm(), dempster_rule(), yager_rule(), murphy_rule(), show_er_result(), run_algorithm_from_file(), and multi_level_multi_source(), which can meet different user demands.

#### er_algorithm()

The er_algorithm() function implements the ER approach and can be called to do multi-source evidence fusion.

ertool.er.er_algorithm(W, DBF, numOfEvidence, numOfPropositions)

Input: *W*, *DBF*, *numOfEvidence*(*nE*), and *numOfPropositions*(*nP*).

Output: *B* (Array) or *False* (Boolean). Their specific meanings have been described in the previous section of programming implementation in Algorithm 1.

#### dempster_rule()

The dempster_rule() function implements the original Dempster’s combination rule to do multi-source evidence fusion. The output of the dempster_rule() function is the same as the er_algorithm() function, while its input does not require the weight input *W*.

ertool.er.dempster_rule(DBF, numOfEvidence, numOfPropositions)

#### yager_rule()

The yager_rule() function implements the Yager’s combination rule [18] to do multi-source evidence fusion. The output of the yager_rule() function is the same as the dempster_rule() function.

ertool.er.yager_rule(DBF, numOfEvidence, numOfPropositions)

#### murphy _rule()

The murphy_rule() function implements the Murphy’s combination rule [19] to do multi-source evidence fusion. The output of the murphy_rule() function is the same as the dempster_rule() function.

ertool.er.murphy_rule(DBF, numOfEvidence, numOfPropositions)

#### show_er_result()

The show_er_result() function helps to visualize the computational results of the ER algorithm.

ertool.er.show_er_result(B, P)

Input:

• *B*: A one-dimensional array of floats, which is the final combined belief degree array generated by the ER algorithm.
• *P*: A list of strings, which are actually the list of proposition names. If *P* is none, the visualization figure displays the specific proposition name as “Proposition *i*” (*i* = 1, 2, …, *nP*).

Output:

• A bar chart of belief degrees distributed on all propositions and overall uncertainty.

#### run_algorithm_from_file()

The run_algorithm_from_file() function reads data from Comma-Separated Values (CSV) and Microsoft Office Excel (XLSX) files and performs multi-source evidence fusion based on the data using the ER approach, Yager’s combination rule, Murphy’s combination rule, or Dempster’s combination rule.

ertool.er.run_algorithm_from_file(file_path, algorithm)

Input:

• *file_path*: A string. The location of the CSV or XLSX file. Note that the data format strictly follows the format of the provided template.
• *algorithm*: “ER,” “Demp,” “Yager,” or “Murphy.” “ER” stands for the ER approach, “Demp” stands for the Dempster’s combination rule, “Yager” stands for the Yager’s combination rule, and “Murphy” stands for the Murphy’s combination rule. The default algorithm is “ER.” If we select “Demp,” “Yager,” or “Murphy” as the algorithm, the weight in the template file will be ignored.

Output:

• Similar to the functions of er_algorithm() and show_er_result(), it outputs the *B* array and visualized results.

#### multi_level_multi_source()

The multi_level_multi_source() function can perform multi-level multi-source evidence fusion based on data files in folders with a tree-like directory, which has the same hierarchy structure as the multi-level multi-attribute assessment framework.

ertool.er.multi_level_multi_source(folder_path)

Input:

• *folder_path*: A string. The folder path of the files containing data that represent the multi-level multi-source evidence and stored in a fixed data format.

Output:

• The result of multi-level multi-source evidence fusion is generated after combining all evidence data stored in the leaf folders and written into the “Objects_combined.csv” file in the root folder.

### The online version of the ERTool

**Fig. 1.**
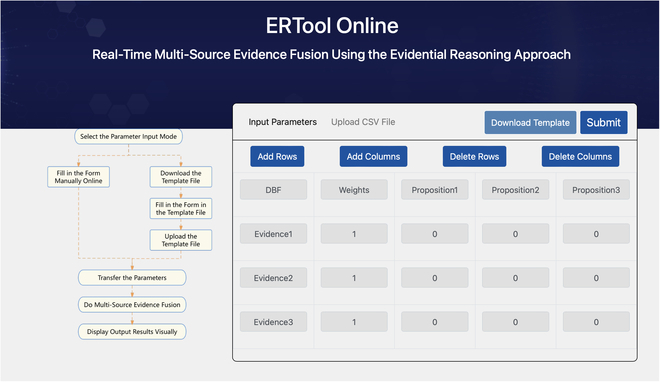
ERTool Online: An online tool for multi-source evidence fusion using the ER approach.

Furthermore, we developed an online multi-source evidence fusion system called ERTool Online (Fig. 1) based on the ERTool Python package. The online ERTool is developed using the Flask framework, where the front end and back end are built. On the front end, the program uses cutting-edge technologies such as JavaScript and Bootstrap to build a highly interactive and user-friendly interface. These technologies not only make web page layouts responsive and aesthetic but also enable effective interaction with users through JavaScript’s dynamic scripting and event-handling mechanisms. Users can enter relevant parameters through this interface intuitively, or directly upload data tables to perform real-time evidence fusion in batch mode. On the back end, the program uses Python programming language and Flask framework to build a solid server-side architecture. Flask’s lightweight and flexibility enable the back end to efficiently process requests from the front end and perform ER-based algorithmic calculations. Python’s powerful data processing capabilities, combined with third-party libraries, such as numpy and pandas, enable fast data processing and analysis on the back end. This web tool realizes the calculation related to ER by calling the ERTool and utilizes third-party libraries at the back end to make the calculation results visualized through vertical bar charts by chart.js.

ERTool’s architecture is crafted to enhance user interaction through clean, intuitive interfaces that simplify the complex process of the ER approach. Moreover, the back end is optimized for high computing efficiency, enabling rapid data processing and analysis without compromising on performance. Accessibility is facilitated by deploying the tool as an open-source resource, readily available for adaptation and use across diverse operational environments.

ERTool Online combines the front end’s interactivity with the back end’s computing power to provide users with an intuitive and efficient online multi-source evidence fusion tool. Through this platform, users can easily upload and process data, and get the results of ER-based multi-source evidence fusion in real time.

## Use Instruction and Examples

### Single-level multi-source evidence fusion

**Fig. 2.**
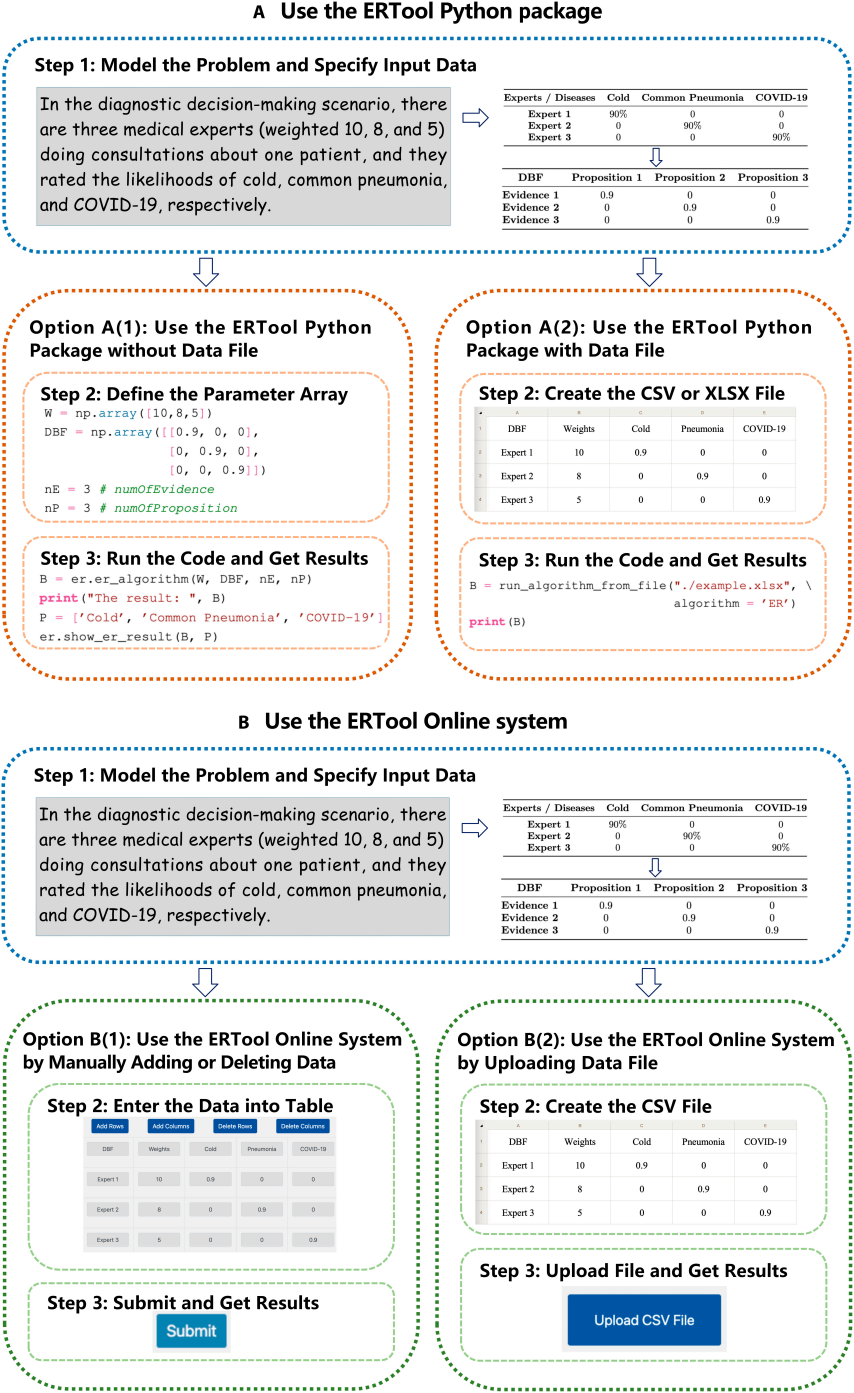
Options for using ERTool to do multi-source evidence fusion. (A) Use the ERTool Python package. (B) Use the ERTool Online system.

The ERTool is compatible with Python 3 and relies on the numpy, pandas, and matplotlib packages [20]. ERTool performs well with numpy version 1.11.3 or higher, pandas version 0.19.2 or higher, and matplotlib version 2.0.0 or higher. In either the ERTool Python package or the web-based ERTool Online system, there are 2 optional ways for calling the ERTool to do multi-source evidence fusion. Detailed procedures for utilizing ERTool in the 2 different system environments are shown in Fig. 2.

As shown in Fig. 2, a complete ER-based evidence fusion process in both the local and online systems is illustrated with a simple example. In the diagnostic decision-making scenario, 3 medical experts (weighted 10, 8, and 5) are doing consultations about one patient, and they rate the likelihood of cold, common pneumonia, and COVID-19, respectively. The inputs of the 3 experts are shown in Table 1.

**Table 1.** An example of ER-based multi-source evidence fusion

Experts	Diseases		
	Cold	Common pneumonia	COVID-19
Expert 1	90%	0	0
Expert 2	0	90%	0
Expert 3	0	0	90%

#### Step 1: Model the problem and specify input data

First, we need to clarify the specific propositions for assessing or evaluating target objects and provide evidence. In the above-mentioned example, each possible disease is set as a proposition, and each medical expert’s diagnosis of the patient is considered as a piece of evidence. Therefore, the above diagnostic decision-making problem can be modeled as the fusion of 3 pieces of evidence with 4 propositions to be assessed. The DBF matrix is shown in Table 2. The weights of the 3 pieces of evidence are 10, 8, and 5, respectively. The ERTool can normalize the weights automatically.

**Table 2.** Degree of belief matrix

DBF	Proposition 1 (cold)	Proposition 2 (common pneumonia)	Proposition 3 (COVID-19)
Evidence 1	0.9	0	0
Evidence 2	0	0.9	0
Evidence 3	0	0	0.9

#### Option A: Use the ERTool Python package.

The first way to apply ERTool in multi-source evidence fusion is to use the ERTool Python package.

#### Step 2: Define the parameter array or create the file

First, we use pip to install the ERTool Python package.

pip install ertool

Then, we have 2 options for multi-source evidence fusion. We can define evidence parameters such as *W* and *DBF* as a numpy array, then call er_algorithm() to run, and call show_er_result() to see the results [option A(1) in Fig. 2]. Alternatively, we can put the data of pieces of evidence into a CSV or XLSX file and call the run_algorithm_from_file() to get the results directly in a visualized way [option A(2) in Fig. 2].

Step 3: Run the ERTool code to get results

Through coding, we can calculate the probability (degree of belief) assigned to each possible disease by using the ER algorithm to fuse evidence.

**Fig. 3.**
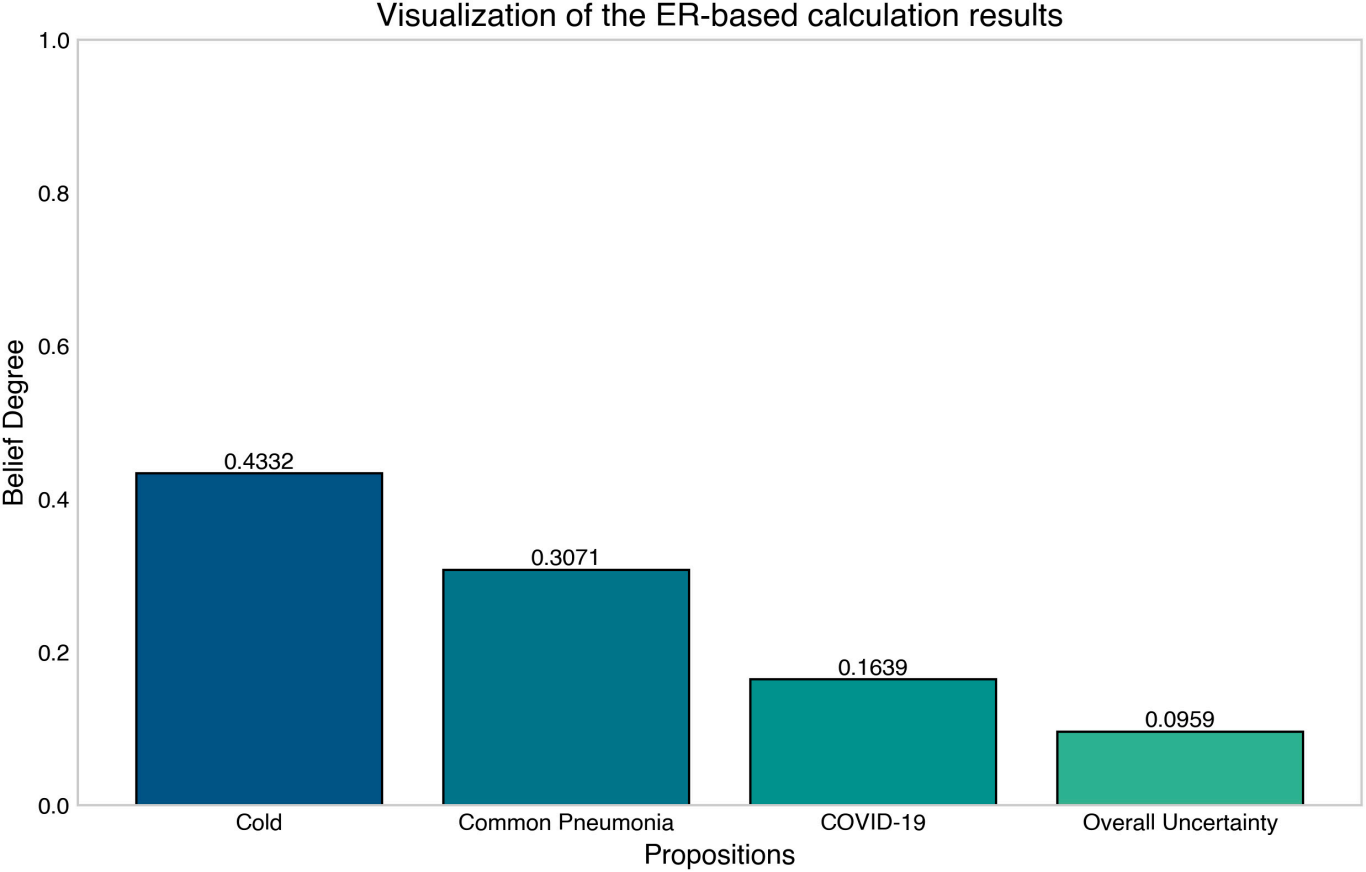
Visualization of the results generated by the ERTool package.

The calculation results show that the probability of the patient being diagnosed with cold, common pneumonia, and COVID-19 is 0.4332, 0.3071, and 0.1639, respectively. The last member of the output *B* represents overall uncertainty, which is 0.0959 in this example. Visualization of the calculation results is shown in Fig. 3.

##### Option B: Use the ERTool Online system.

**Fig. 4.**
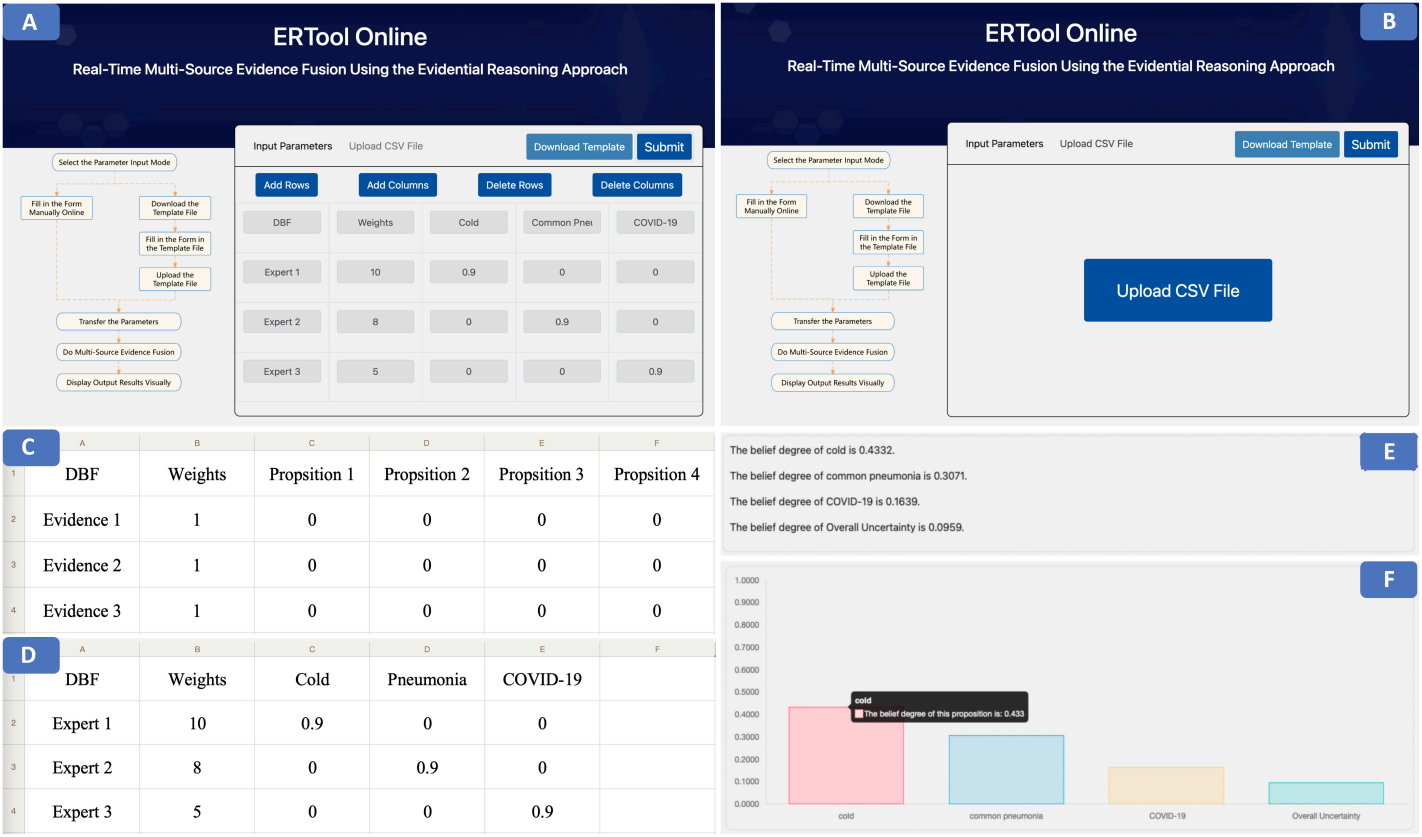
Multi-source evidence fusion using ERTool Online. (A) On the ERTool Online default page, the DBF matrix data can be added or deleted manually [step 2 of option B(1) in Fig. 2]. (B) The DBF matrix data can be inputted by uploading a CSV file [step 2 of option B(2) in Fig. 2]. (C) Template for formatting the DBF matrix. (D) Write the DBF data to the CSV template file. (E) Results of the belief degree assessed to each proposition after evidence fusion, arranged in rows. (F) Online visualization of the evidence fusion results.

We can also use ERTool Online for multi-source evidence fusion (Fig. 4). There are 2 ways to use the online tool. One way [option B(1) in Fig. 2] is to use the ERTool Online by manually adding or deleting data, and the other option [option B(2) in Fig. 2] is to use the ERTool Online by uploading a data file. In either option, we need to enter inputs into the system by online editing or uploading files (step 2). Then, we can click the Submit button to call the backend ERTool, and the calculation results will be visually displayed on the webpage in real time (step 3).

### Multi-level multi-source evidence fusion

The ERTool Python package provides a multi_level_multi_source() function for fusing multi-level multi-source evidence. For all the objects to be assessed, we can store multi-level evidence data in nested folders first, and the folder is structured using the hierarchical multi-level multi-attribute assessment framework. The folder and the evidence data files are built as follows. First, set the name of the root folder as “Objects.” It is the root of the directory tree, and the data file (Objects*_*combined.csv) containing the final combined results of all evidence is put in this folder. Initially, users can enter the names of all objects and propositions into the first column and first row in the file (Objects*_*combined.csv), respectively, and set the initial values of their DBF matrix to zeros. Second, set subfolders corresponding to each sub-attribute at each middle layer, and put a file “*sub*-*attribute_*combined.csv” in each subfolder. It is for storing the combined belief degree in each proposition after fusing all evidence available in assessing this sub-attribute, and the weight column in the file stores the weight of the corresponding sub-attribute. Third, data files containing information on pieces of evidence at a leaf node, named “*evidence*.csv,” are put in the corresponding leaf folder. Note that the data files described here are for storing data of all objects to be assessed.

**Fig. 5.**
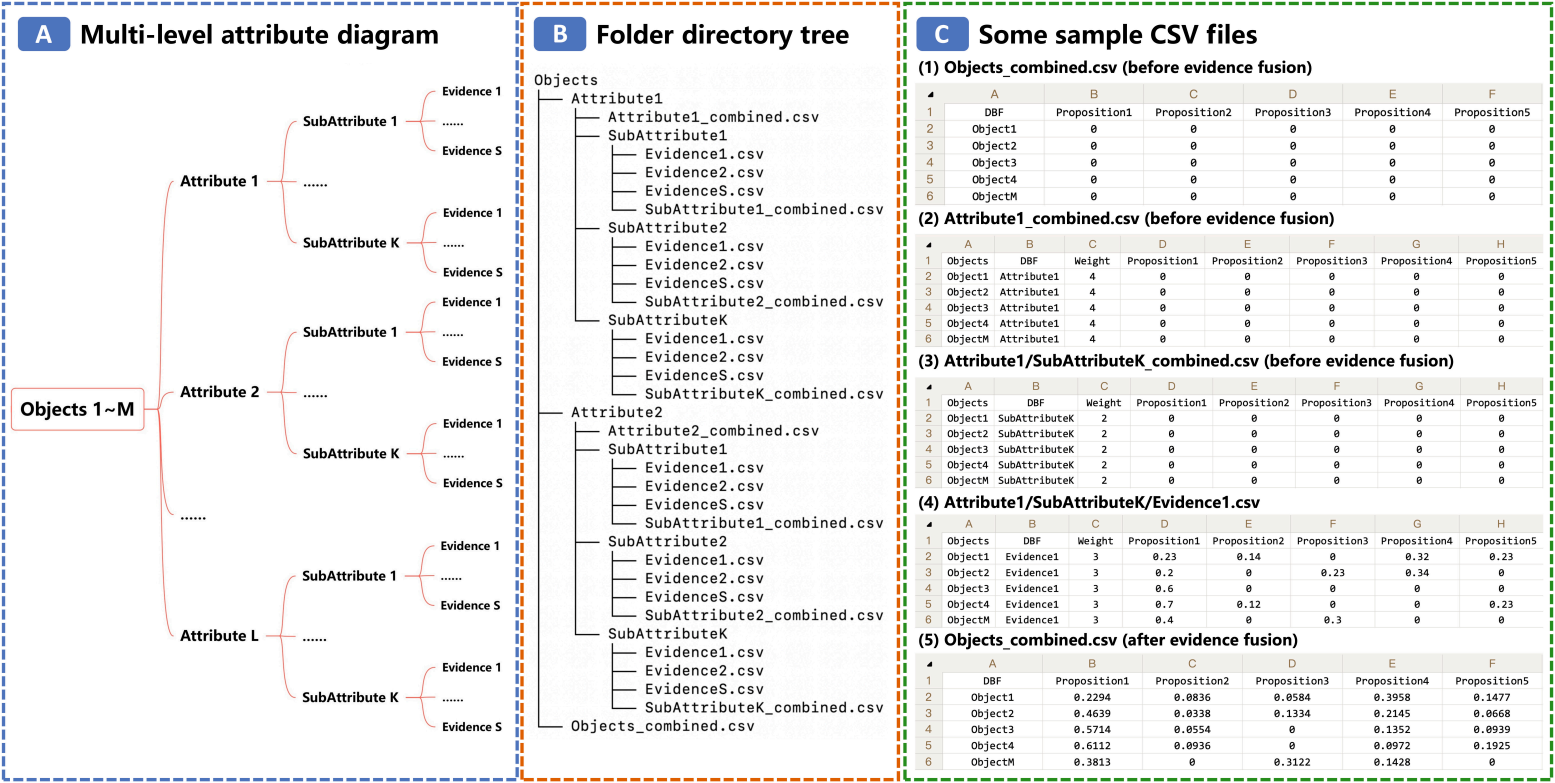
Multi-level multi-source evidence fusion using ERTool Python package. (A) Multi-level multi-attribute evaluation framework. (B) Objects folder with a tree-like directory structure. (C) Exemplar data files before and after evidence fusion.

For example, we define a 2-level multi-attribute assessment framework in Fig. 5A and use the tree-like Objects folder to illustrate the problem evaluation framework and store related evidence data. The directory structure of the Objects folder and corresponding data files are shown in Fig. 5B. We can perform 2-level multi-source evidence fusion based on all evidence-related data files stored in the Objects folder by calling the multi_level_multi_source() function.

As shown in Fig. 5C, the combined results of the exemplar 2-level multi-source evidence generated by the ERTool for the *M* objects are written into the “Objects*_*combined.csv” file in the root folder.

**Fig. 6.**
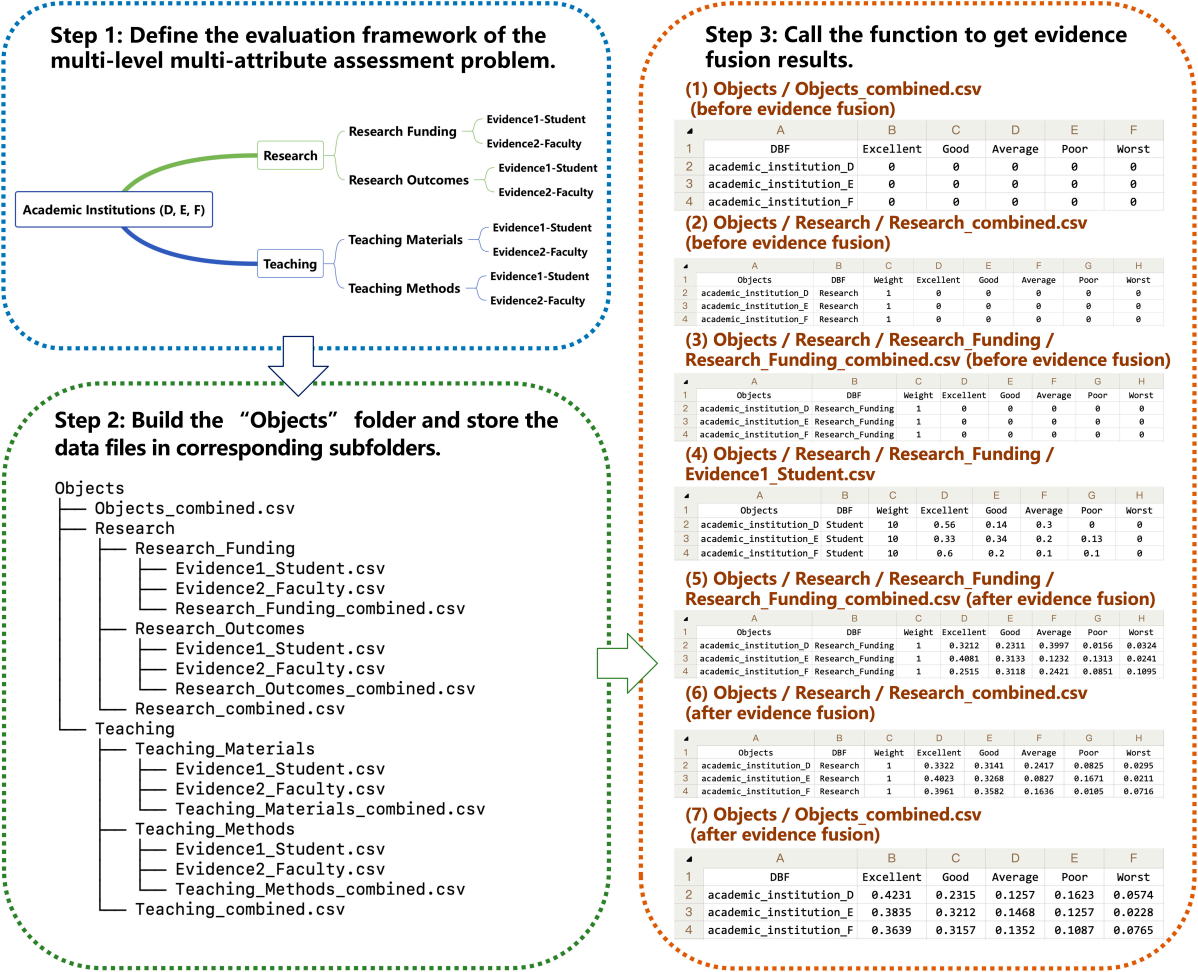
An example of using ERTool for multi-level multi-source evidence fusion.

Suppose 3 academic institutions (D, E, F) are evaluated using a 2-level and multi-attribute evaluation framework. Assume the 3 institutes are evaluated from research and teaching perspectives, while research is assessed based on research funding and research outcomes, and teaching is evaluated based on teaching materials and teaching methods. The evaluation is conducted by surveying both students and faculties. The evaluation grades include Excellent, Good, Average, Poor, and Worst. Assuming that student assessments are more important than faculty judgments, we give a weight of 10 to student feedback and 8 to faculty evaluation. Meanwhile, we consider the weight of each assessment attribute equal and be 1. The detailed procedures for using the multi_level_multi_source() function are shown in Fig. 6.

#### Step 1: Define the evaluation framework of the multi-level multi-attribute assessment problem

First, we define the above 2-level multi-attribute assessment problem using the ER framework. The 2-level multi-attribute evaluation framework can be illustrated using the diagram as shown in Fig. 6 (step 1).

#### Step 2: Build the “Objects” folder and store the data files in corresponding subfolders

Second, we build the “Objects” folder and its subfolders according to the evaluation framework. Save the survey data about each attribute (research funding, research outcomes, teaching materials, teaching methods) of the 3 institutes from surveyed students and faculties together with corresponding weight to different CSV files. Put different evidence data files (Evidence1_Student.csv and Evidence2_Faculty.csv) and the data files for storing evidence combined results (Objects_combined.csv, Research_combined.csv, Research_Funding_combined.csv, Reseach_Outcomes_combined.csv, Teaching_combined.csv, Teaching_Materials_combined.csv, Teaching_Methods_combined.csv) to their corresponding subfolders, as illustrated in Fig. 6 (step 2).

#### Step 3: Call the function to get evidence fusion results

Finally, we call the multi_level_multi_source() function using the path of the “Objects” folder as input, and the output results will be written to “Objects_combined.csv” file by running the following codes:

from ertool import er

er.multi_level_multi_source(*)

Here, “*” represents the path of “Objects” folder. For example, it can be “/Users/tyshi/Documents/Objects.”

## Validation and Comparison

We validated the ERTool and compared it with the ER-based IDS software in dealing with a specific evidence fusion task for medical quality assessment. The IDS is a Windows-based software package developed for handling multiple criteria decision analysis (MCDA) problems with uncertainty. It is based on the ER approach. IDS models MCDA problems with different criteria using a belief decision matrix, which represents pieces of evidence with different belief degrees distributed on different propositions or evaluation grades for each criterion.

We used both ERTool and IDS software to do evidence fusion for combining patient feedback on the medical quality of 3 hospitals from medical facilities (MFs), medical staff (MS), medical processes (MPs), and medical outcomes (MOs) perspectives. The summary statistics of patient feedback on the medical quality of hospitals A, B, and C from the 4 different perspectives are shown in Table 3. Taking the assessment of MF of hospital A as an example, data of the first row corresponding to hospital A show that 19.75% of patients rank the hospital’s facilities as excellent, 37.04% as good, 39.51% as average, 2.47% as poor, and only 1.23% consider the facilities as worst.

**Table 3.** Patient feedback on medical quality of hospitals A, B, and C

Hospitals	Quality items	Excellent	Good	Average	Poor	Worst
Hospital A	MF	19.75%	37.04%	39.51%	2.47%	1.23%
	MS	18.52%	39.51%	35.80%	3.70%	2.47%
	MP	12.35%	33.33%	49.38%	4.94%	0.00%
	MO	14.81%	48.15%	30.86%	6.17%	0.00%
Hospital B	MF	19.72%	53.52%	23.94%	2.82%	0.00%
	MS	19.72%	49.30%	28.17%	2.81%	0.00%
	MP	19.72%	45.07%	35.21%	0.00%	0.00%
	MO	21.13%	47.89%	28.17%	2.81%	0.00%
Hospital C	MF	23.68%	50.00%	22.37%	2.63%	1.32%
	MS	26.32%	47.37%	21.05%	5.26%	0.00%
	MP	19.74%	43.42%	31.58%	3.95%	1.31%
	MO	27.63%	46.05%	23.68%	1.32%	1.32%

**Fig. 7.**
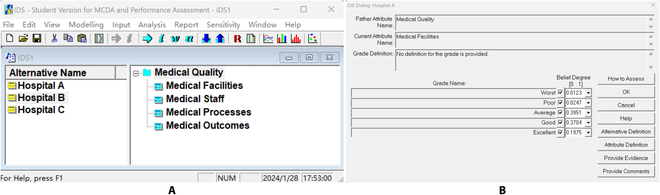
Modeling the medical quality assessment problem in IDS. (A) IDS main window for modeling the problem. (B) IDS dialog for inputting belief degrees in different assessment propositions.

**Fig. 8.**
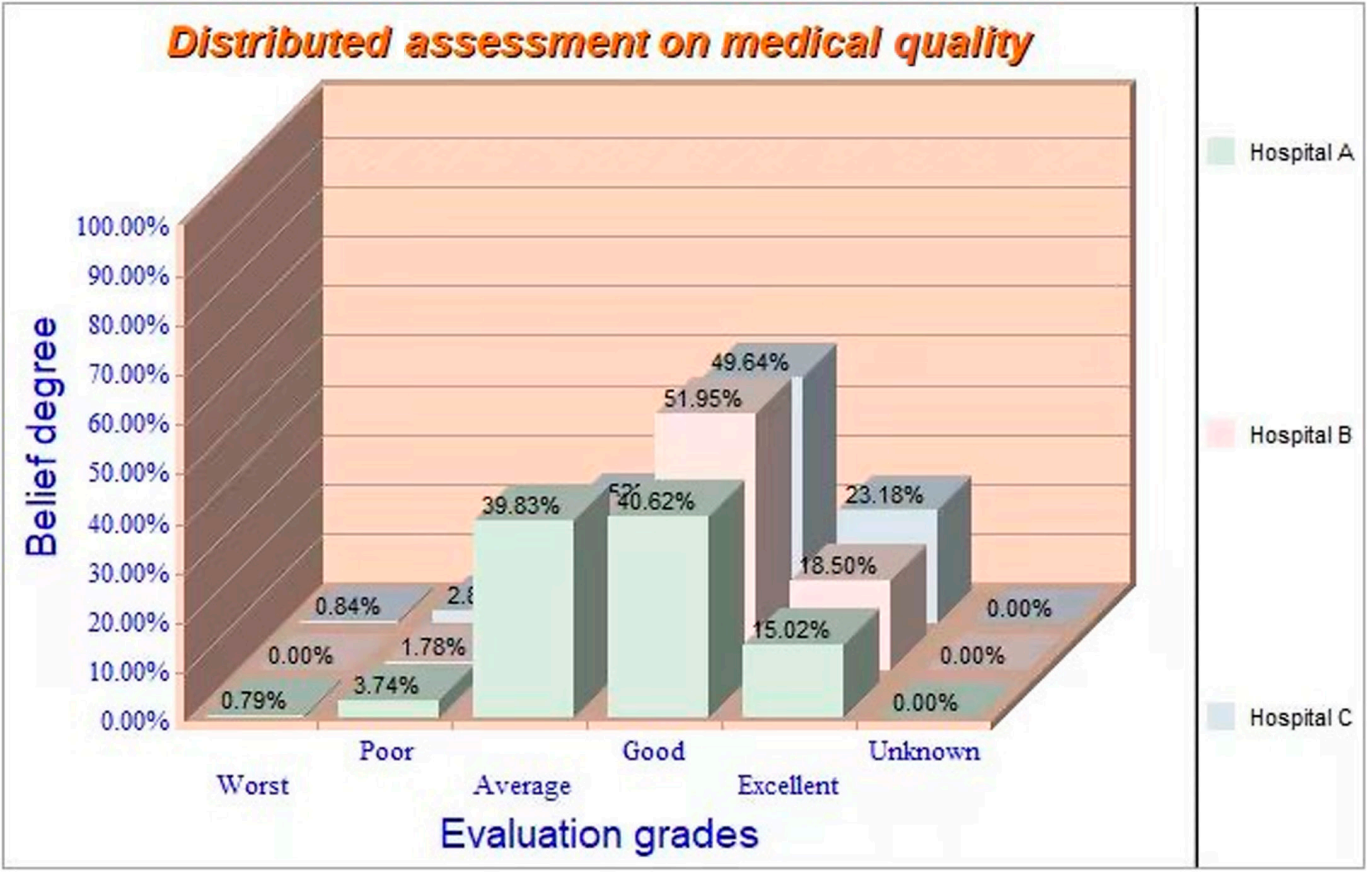
Evidence fusion results generated by IDS.

If we use IDS to deal with the medical quality assessment problem, Fig. 7 shows the interfaces for modeling the problem and inputting belief degrees in all propositions for each quality criterion in IDS, and Fig. 8 illustrates the combined belief degrees in different propositions for all the 3 hospitals after evidence fusion by the IDS.

**Fig. 9.**
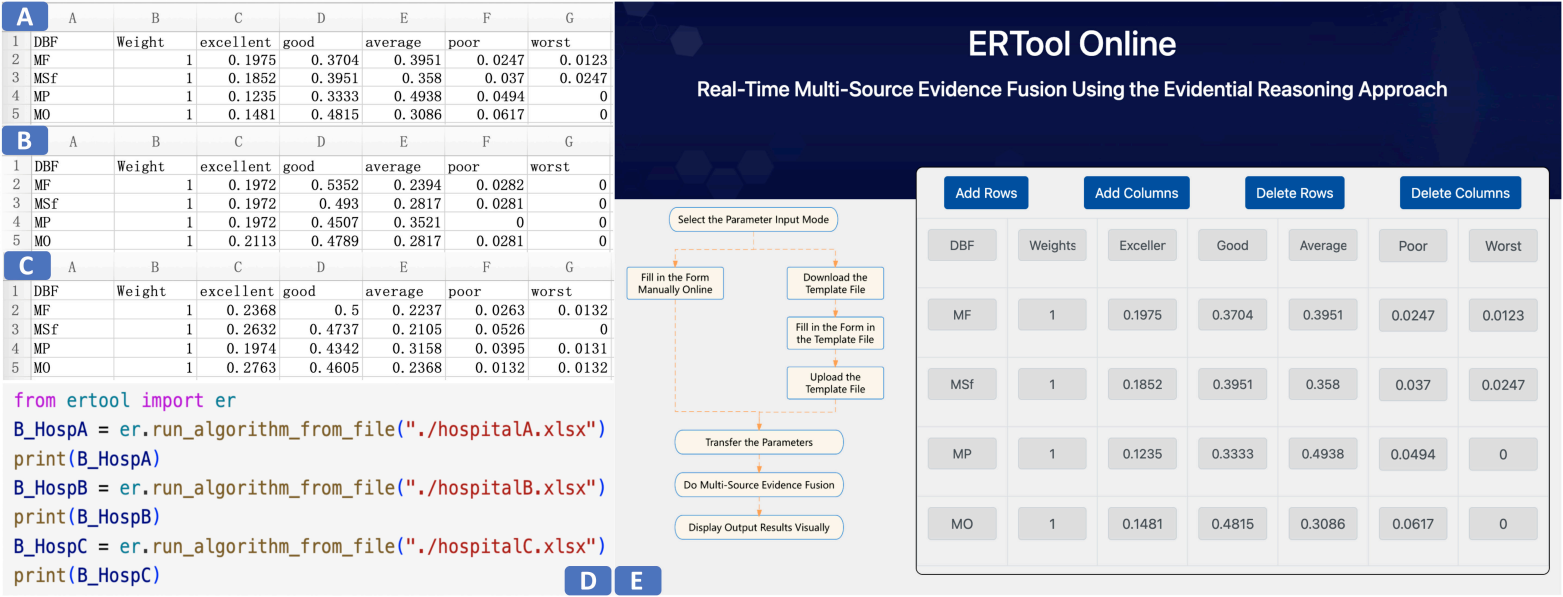
The interfaces for inputting DBF data in local or online ERTool. (A to C) DBF data file of hospitals A, B, and C. (D) Run ERTool using Python code. (E) Data entry using ERTool Online.

If we utilize the ERTool to handle the medical quality assessment problem, we do not need to model the problem in the local or online tool; what we need is to input DBF data and call the tool. The interfaces for inputting DBF data in local and online ERTool are shown in Fig. 9, and the evidence fusion results generated by the ERTool are shown in Table 4.

**Table 4.** The evidence fusion results generated by the ERTool

Hospitals	Excellent	Good	Average	Poor	Worst
Hospital A	0.1502	0.4062	0.3983	0.0374	0.0079
Hospital B	0.1850	0.5195	0.2777	0.0178	0
Hospital C	0.2318	0.4964	0.2352	0.0282	0.0084

We found that the results calculated by ERTool were the same as the results generated by IDS software. It validated the accuracy and reliability of ERTool in conducting ER-based multi-source evidence fusion jobs.

Furthermore, we compared the ERTool and IDS from different angles. As shown in Table 5, we find that ERTool has several advantages over IDS. First, the Python-based interfaces of ERTool provide better user-friendliness, especially beneficial for those familiar with Python programming. Compared to the interfaces for manual decision problem modeling in IDS, the interface for problem modeling and data input is much simpler and intuitive in ERTool. But we notice that the IDS tool can provide visualized modeling for hierarchical multi-attribute problems and support multi-level multi-source evidence fusion, while the ERTool provides a multi_level_multi_source() function to perform bottom-up evidence fusion based on data files stored in a tree-like folder directory. The ERTool utilizes a static tree-like folder directory to replace the dynamic and visualized hierarchical multi-attribute modeling process in IDS, and it uses electronic data files other than manual data inputting to implement system input.

**Table 5.** Comparison of IDS software and ERTool Python package

Comparison aspect	IDS software	ERTool
User friendliness	Multiple menus with different interfaces, and non-professional users require training for system use.	Simple interfaces and intuitive system usage, user-friendly for non-professional users.
Open-source	Not open-source, and system installation is required.	Open-source, and freely accessible to any user.
System scalability	System functionality is limited to its own environment.	System functionality can be easily extended within Python ecosystem.
Data input mode	Data need to be inputted manually step by step.	Data can be inputted manually or uploaded automatically.
Function customization	System functions are fixed.	Allowing users to view, modify, and optimize the codes of any function.
Operating system environment	Only Windows.	Windows, Mac OS, and Linux.

Second, the ERTool allows for uploading a data file and it can deal with multi-source evidence fusion in a batch mode. Note that the ERTool has the flexibility to handle various data formats, including CSV and XLSX files, and provides a smoother data processing experience.

Third, the open-source nature is the key advantage of ERTool, and it provides free accessibility and code customization capabilities to the user community. This helps promote the usage of ER in both academic and industry fields. Moreover, its integration within the Python ecosystem enables the ERTool to be seamlessly incorporated into bigger systems, while extending the functionalities of IDS is a little bit difficult and it is limited to its current version.

**Fig. 10.**
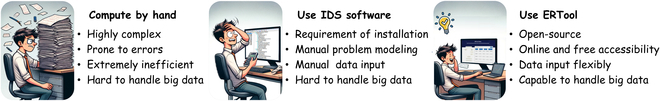
Characteristics of multi-source evidence fusion provided by different tools.

Figure 10 illustrates the improvement of ER-based evidence fusion from hand computation to IDS and to ERTool.

To summarize, the ERTool can not only provide evidence fusion functionality just like the ER-based IDS but also bring substantial improvements in terms of ease of use, flexibility in data processing, and free accessibility. These advantages and improvements make ERTool highly valuable and functional for researchers and practitioners in evidence-based decision-making, enhancing the efficiency and scope of their domain work. This positions ERTool not only as a reliable alternative to the existing IDS but also as a preferred tool for future research and application of multi-source evidence fusion.

## Conclusion and Future Work

In this study, we designed and developed the ERTool, a Python package that implements the ER approach. Meanwhile, we also developed a web-based system ERTool Online. The online tool can aid decision-makers in merging multi-source evidence by inputting data manually or uploading data files automatically, and it can generate ER-based calculation results in real time. Compared to the existing ER-based decision support tools such as IDS, the ERTool has many advantages, including open-source, free accessibility, clean interfaces, and high computing efficiency. These characteristics ensure that the tool not only meets the current demands of ER applications but also sets a new standard for usability and accessibility in decision support systems. Although ERTool has demonstrated its capabilities in supporting multiple evidence-based decision-making, there is still potential for enhancement. Currently, ERTool utilizes a folder directory and CSV file management system for multi-level multi-source evidence fusion, which holds evidence data with regular volumes. To handle evidence with large volumes, we plan to integrate database management systems (DBMSs) into ERTool, aiming to improve its scalability and performance. More specifically, we will adopt a structured approach to better manage and store evidence data within the DBMS, where we will create specific tables for various types of information. These will include *Evidence Table* for storing each piece of evidence, containing information like the evidence source and collection date; *Propositions Table* for documenting potential outcomes or evaluation grades associated with the evidence; and *Evidence-Propositions Table* that links each piece of evidence to its possible outcomes or evaluation grades, recording the associated belief degrees. This interconnected table structure will allow us to retrieve and combine evidence for different decision-making jobs efficiently. In the next step, we will study the feasibility of the proposed evidence data management plan. Moreover, although the ER approach supports the power set of the frame of discernment theoretically, the traditional complete set of the discernment frame works well in using the ER approach to support multiple evidence-based decision-making in practice, and thus, the current version of ERTool supports only the complete set of discernment frame. At the same time, because the actual evidence is generally reliable, it is assumed that each piece of evidence has the same reliability, and thus, we considered only the weight of each piece of evidence in the developed ERTool. In the future, we will consider the power set of the frame of discernment and evidence reliability in the next version of ERTool. We are committed to the continuous improvement of ERTool by integrating the latest advancements in the ER approach. This will not only ensure its ongoing relevance and superiority in the field but also broaden its capabilities to include increasingly sophisticated models as they emerge. To foster innovation and adaptability, we plan to establish a community-driven update framework, allowing other researchers and practitioners to contribute to the ERTool’s evolution actively. Hopefully, the ERTool will be a key tool for efficient multi-source evidence fusion in a variety of complex decision-making scenarios.

## Data Availability

ERTool is compatible with Python 3 and can be installed through the Python Package Index (PyPI) at https://pypi.org/project/ERTool/ or https://github.com/STYAI/ERTool. We can read documents of ERTool and use ERTool Online by visiting https://www.ertool.online.
